# Quantification of arterial, venous, and cerebrospinal fluid flow dynamics by magnetic resonance imaging under simulated micro-gravity conditions: a prospective cohort study

**DOI:** 10.1186/s12987-021-00238-3

**Published:** 2021-02-12

**Authors:** Arslan M. Zahid, Bryn Martin, Stephanie Collins, John N. Oshinski, C. Ross Ethier

**Affiliations:** 1 Emory University School of Medicine, Atlanta, GA USA; 2 Department of Biological Engineering, University of Idaho, Moscow, Idaho USA; 3 Department of Biomedical Engineering, Georgia Institute of Technology and Emory University School of Medicine, Atlanta, GA USA; 4 Department of Radiology and Imaging Sciences, Emory University, Atlanta, GA USA; 5 Alycone Therapeutics, Lowell, MA USA; 6 University of Chicago, 900 S Clark Street, Apt 1001, Chicago, IL 60605 USA

**Keywords:** Spaceflight‐associated neuro‐ocular syndrome, Head‐down tilt, Arterial, venous, cerebrospinal fluid dynamics, Simulated microgravity

## Abstract

**Background:**

Astronauts undergoing long-duration spaceflight are exposed to numerous health risks, including Spaceflight-Associated Neuro-Ocular Syndrome (SANS), a spectrum of ophthalmic changes that can result in permanent loss of visual acuity. The etiology of SANS is not well understood but is thought to involve changes in cerebrovascular flow dynamics in response to microgravity. There is a paucity of knowledge in this area; in particular, cerebrospinal fluid (CSF) flow dynamics have not been well characterized under microgravity conditions. Our study was designed to determine the effect of simulated microgravity (head-down tilt [HDT]) on cerebrovascular flow dynamics. We hypothesized that microgravity conditions simulated by acute HDT would result in increases in CSF pulsatile flow.

**Methods:**

In a prospective cohort study, we measured flow in major cerebral arteries, veins, and CSF spaces in fifteen healthy volunteers using phase contrast magnetic resonance (PCMR) before and during 15° HDT.

**Results:**

We found a decrease in all CSF flow variables [systolic peak flow (p = 0.009), and peak-to-peak pulse amplitude (p = 0.001)]. Cerebral arterial average flow (p = 0.04), systolic peak flow (p = 0.04), and peak-to-peak pulse amplitude (p = 0.02) all also significantly decreased. We additionally found a decrease in average cerebral arterial flow (p = 0.040). Finally, a significant increase in cerebral venous cross-sectional area under HDT (p = 0.005) was also observed.

**Conclusions:**

These results collectively demonstrate that acute application of −15° HDT caused a reduction in CSF flow variables (systolic peak flow and peak-to-peak pulse amplitude) which, when coupled with a decrease in average cerebral arterial flow, systolic peak flow, and peak-to-peak pulse amplitude, is consistent with a decrease in cardiac-related pulsatile CSF flow. These results suggest that decreases in cerebral arterial inflow were the principal drivers of decreases in CSF pulsatile flow.

## Background

Microgravity has been associated with multiple maladaptive physiological processes, including alterations to cerebrospinal fluid (CSF) flow and pressures in microgravity. CSF production and its flow characteristics serve as a key component of the physiological homeostasis, immunological protection, and metabolic maintenance of the central nervous system. Magnetic resonance imaging (MRI) studies in astronauts before and after spaceflight missions have shown perturbations to the CSF spaces, predominantly after long-duration missions, including narrowing of the central sulcus, upward shift of the brain, and narrowing of the CSF spaces at the vertex [1].

A spaceflight-induced condition thought to be related to alterations in CSF flow dynamics is Spaceflight Associated Neuro-ocular Syndrome (SANS), a spectrum of poorly understood neuro-ophthalmological findings documented in a subset of astronauts returning from the International Space Station. Clinical findings of SANS include unilateral and bilateral optic disc edema, globe flattening, choroidal and retinal folds, hyperopic refractive error shifts, and choroidal and nerve fiber layer thickening [2]. Of seven United States astronauts evaluated for SANS after long-duration spaceflight of greater than six months, six developed decreased near field vision with nerve fiber layer thickening; five exhibited optic disc edema, globe flattening, and choroidal folds; and three had nerve fiber layer thickening [2, 3]. Four individuals who underwent postflight lumbar puncture for measurement of CSF pressure exhibited an elevated average opening pressure of 28 cm H_2_O up to 12 days after reentry [2, 3]. In addition, postflight surveys of 300 astronauts suggest that inflight loss of both near and distant visual acuity is a significant issue that increases in prevalence with duration of flight, with 29 % of short duration and 60 % of long duration mission participants reporting some degree of loss in visual acuity [2, 4].

Alterations in CSF dynamics secondary to loss of hydrostatic pressure gradients in microgravity are thought to play a role in SANS. Such alterations may include an overall mild elevation in intracranial pressure (ICP) and/or a loss of normal CSF pressure changes due to postural changes on Earth; however, the physiology of the interaction between cerebral blood flow and CSF in microgravity is not well understood. Although not conclusively known, the pathology observed in astronauts may suggest transiently increased ICP; according to the “pulse absorber” or “cerebral Windkessel” theory, this should correspond to an increase in CSF flow pulsation [5, 6]. Thus, both cerebral venous and arterial flow, as the principal contributors to changes in CSF flow related to this increased ICP, are physiologically plausible factors affecting CSF flow dynamics, as demonstrated in studies of young adults and elderly subjects by PCMR [7]. Cerebral arterial and jugular venous flow pulsations correlate significantly with CSF flow pulsations, with cerebral arterial flow having a predominantly positive contribution to CSF flow pulsations, opposite to the contribution of jugular venous flow [8]. Thus, a decrease in arterial cerebrovascular inflow would be associated with a decrease in cardiac-related pulsatile CSF flow. It is also notable that work by Roberts et al. suggests that the upward shift of the brain in microgravity with tissue crowding at the vertex may compress adjacent venous structures, causing obstruction of CSF and venous outflow, thereby potentially resulting in an elevation of ICP [1].

Prior studies using ground-based simulated microgravity have demonstrated changes in cerebral arterial and jugular flow dynamics, however changes in CSF pulsations would benefit from improved understanding. The study of cerebral arterial, venous, and CSF flow dynamics as they relate to microgravity would be valuable in their own right as well as being useful for helping to validate whole-body models that predict SANS pathophysiology and seek to evaluate the effectiveness of countermeasures [4, 9, 10].

Head-down tilt (HDT) has been used to simulate microgravity in ground-based studies that seek to quantify changes in arterial and venous flow dynamics [11]. However, prior studies have yielded conflicting results about arterial blood flow dynamics and have not comprehensively studied CSF flow dynamics, which is an important underlying aspect of the “fluids shift” hypothesis for SANS [4, 12, 13].

In the present study, we hypothesized that microgravity conditions simulated by acute HDT would result in increased CSF pulsatile flow. Our study was designed to analyze the effect of HDT simulated microgravity on cerebrovascular flow dynamics by measuring flow through major arteries, veins, and CSF spaces using phase contrast magnetic resonance (PCMR) before and after acute application of 15° HDT in healthy volunteers.

## Materials and methods

### Study design

Fifteen healthy subjects participated in this NASA and Emory University IRB-approved study protocol. This study adhered to the tenets of the Declaration of Helsinki. Criteria for inclusion in the study included age ≥ 18 years, no contraindications to non-contrast MRI, no self-reported cardiovascular or neurological disease, and no current pregnancy. Written informed consent was obtained for all volunteers. All data was anonymized prior to analysis.

### Physiological measurements

Heart rate was monitored throughout the exam using a peripheral pulse unit applied to the first digit of the volunteer’s left hand. Blood pressure readings were taken prior to the exam, in the supine position, and in the HDT position after completion of the MRI scan. Blood pressure readings were taken with a MR-safe digital automated sphygmomanometer applied to the upper arm while the volunteer was supine.

### In‐vivo MRI measurements

**Fig. 1:**
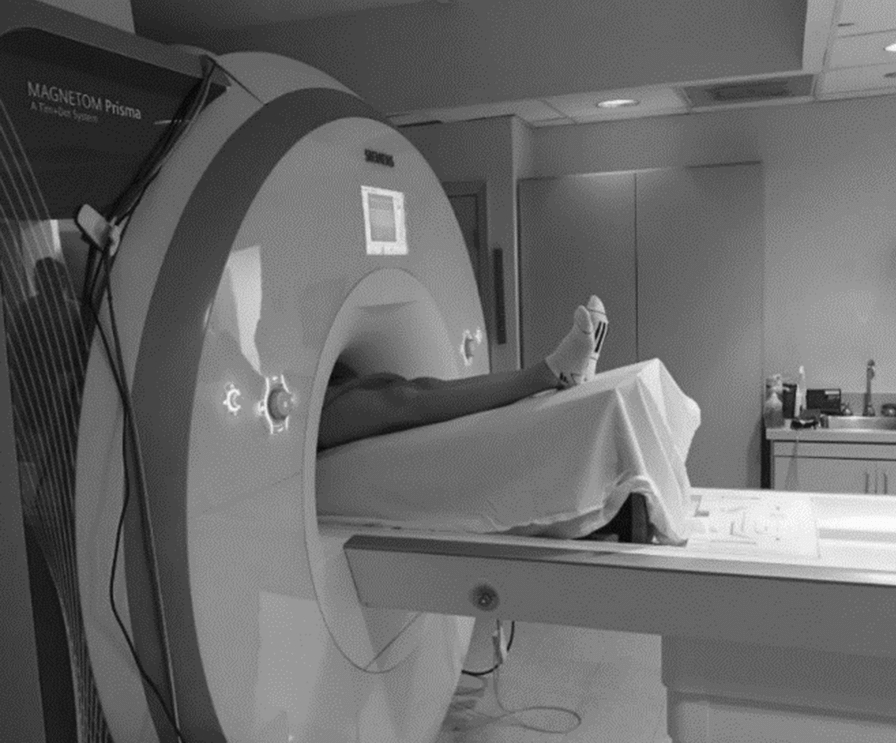
Volunteer under −15° head-down tilt in the MR scanner. Note that the head (not pictured) was positioned within a radiofrequency coil that required it to be positioned at 0° orientation above the neck

All volunteers were scanned on a 3 T MRI scanner (Prisma Siemens Medical Systems, Malvern, PA) using a 20-channel head coil. Two-dimensional cine phase-contrast MRI (PCMR) images were acquired in the transverse orientation at the mid-C2 vertebral level using a previously published protocol [14]. Images were acquired with through-plane velocity encoding in the supine position and again after a 30-minute acclimation period at 15° HDT (Fig. 1). Forty images were reconstructed for each cardiac cycle using retrospective peripheral pulse unit (PPU) gating. Imaging variables included a field-of-view (FOV) = 175 × 175 mm, in-plane resolution = 1.1 × 1.1 mm, slice thickness = 5.0 mm, repetition time (TR) = 11 ms, echo time (TE) = 7 ms, flip angle = 30°, and two segments. Two scans were acquired at the mid-C2 location, one with a velocity encoding (VENC) value of 80 cm/s for blood flow and one with a VENC value of 10 cm/s for CSF flow. The 15° HDT was achieved using a custom-made wedge (Fig. 1). This scan protocol was performed twice for each volunteer, first while supine, and then under HDT following a 30-minute acclimatization period under HDT.

**Fig. 2:**
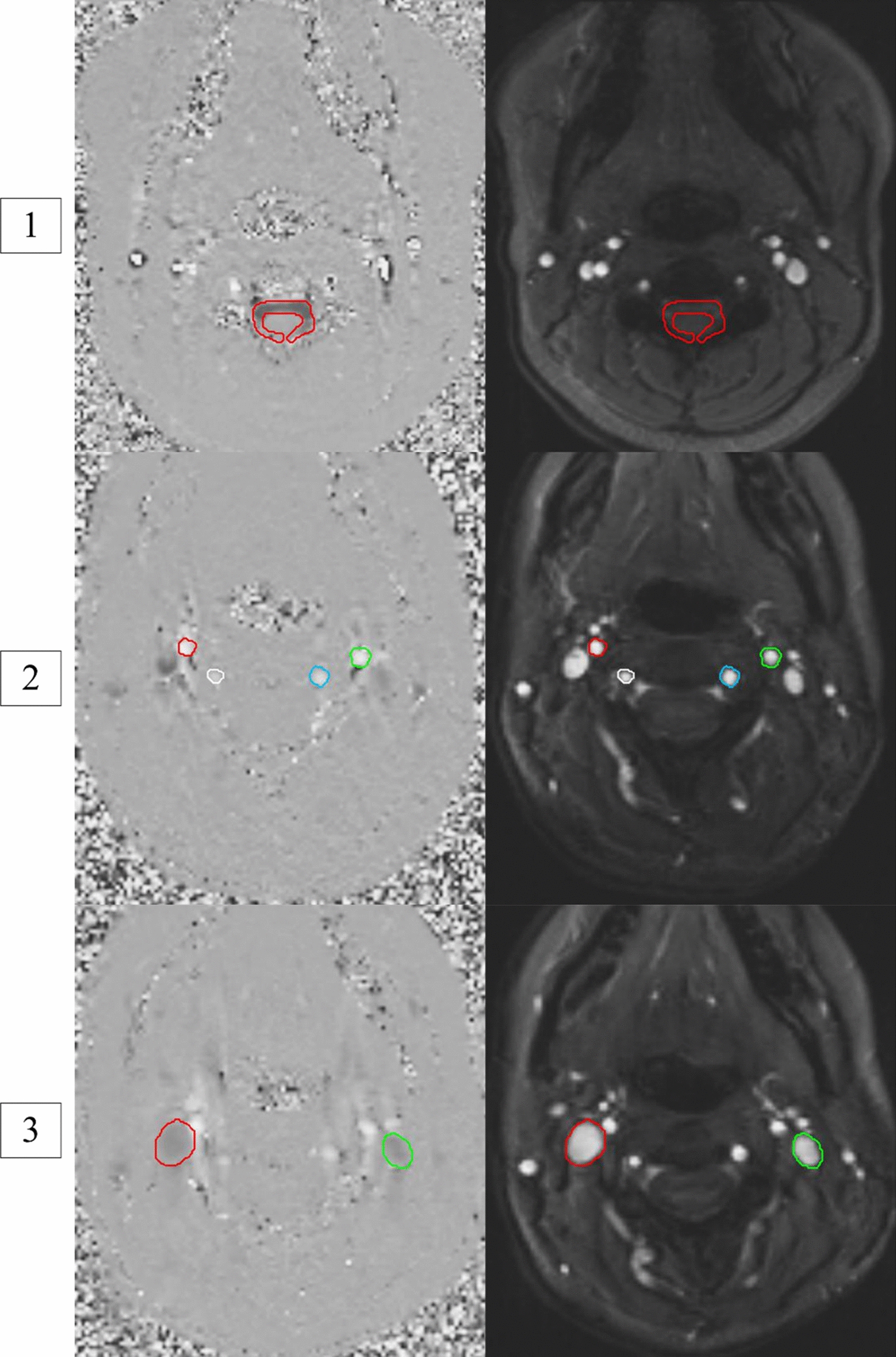
Example of CSF (image 1), arterial (image 2), and venous (image 3) ROIs as seen on an axial view at the mid-C2 vertebral level. Each scan is from a different volunteer. Left greyscale panels: phase contrast image showing craniocaudal velocities (greyscale). Right panels: anatomic image used to identify vessels and other structures. Image 1 structures: subarachnoid space (red). Image 2 structures: internal carotid (red and green) and vertebral arteries (white and blue). Image 3 structures: Internal jugular veins (red and green). Instantaneous flow rates (mL/min) at 40 time points throughout the cardiac cycle were determined in the veins, arteries, and CSF spaces using the ROI area and corresponding average velocity measurements as determined by the FLOW program. The software also provided stroke volumes for each vessel, defined as the net blood volume crossing the measurement plane per cardiac cycle

To quantify CSF, venous, and arterial flow, regions of interest (ROI) were outlined within the subarachnoid space, the bilateral internal jugular veins, and the bilateral internal carotid and vertebral arteries, respectively (Fig. 2). ROIs were drawn manually to approximate the contours of each vessel using FLOW software suite (LUMC, Lieden, The Netherlands) [15, 16]. Each ROI for each of the forty images per scan was visually inspected to ensure quality of the contours. The automatic segmentation feature was applied at each time-step to correctly segment the vessel wall. All ROIs were visually inspected to assure that phase aliasing was not present, and if found, phase unwrapping was applied.

**Fig. 3:**
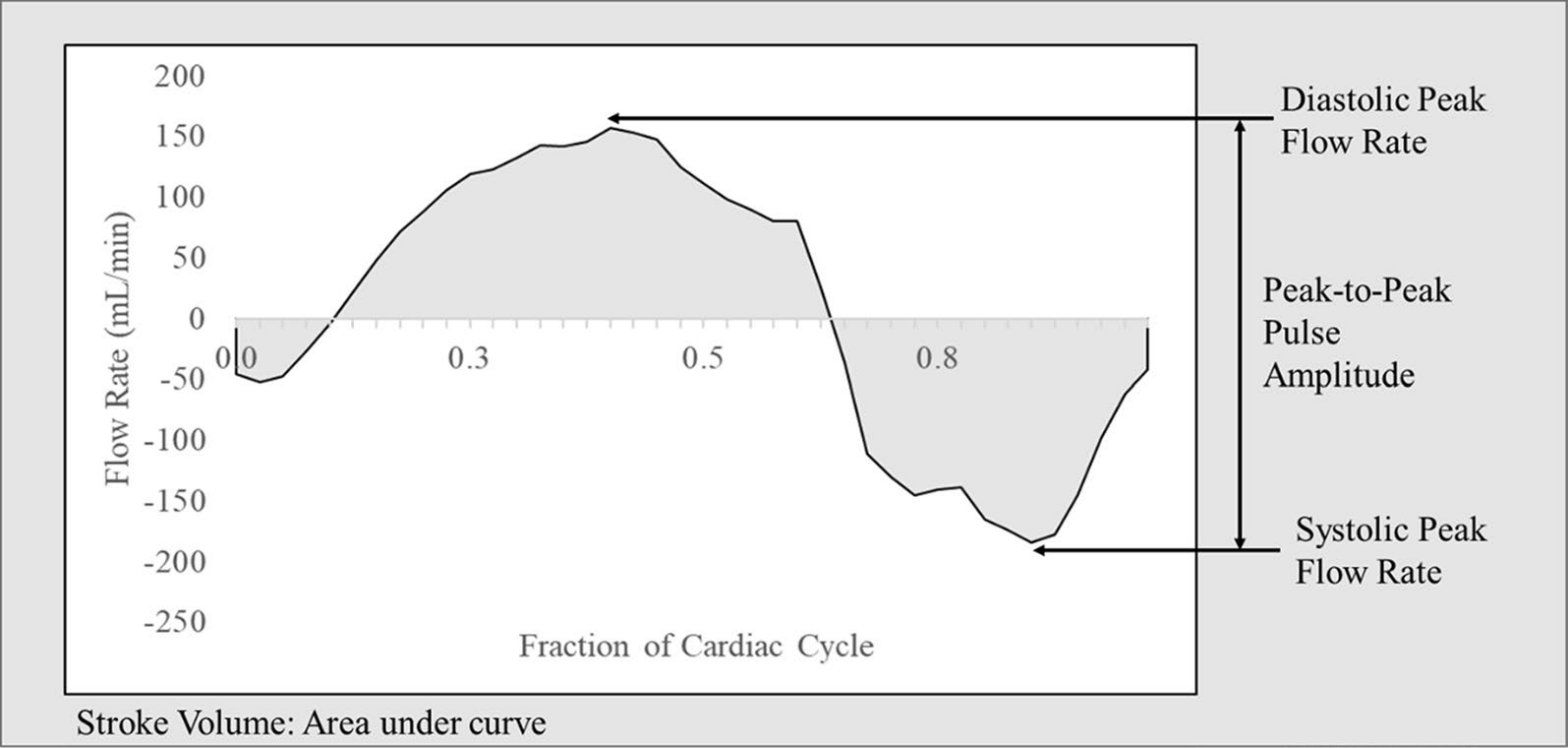
Representative CSF flow rate vs. time over a cardiac cycle, showing the definitions of flow and pulsatility variables. Note that negative flow rates present during systole are in the craniocaudal direction. The pulse was monitored by a peripheral pulse unit applied to the first digit of the volunteer’s hand, and the local systolic flow peak occurs earlier at the vessels of the neck than at the first digit, explaining the location of the systolic peak in this figure. Time zero of the cardiac cycle in this figure represents the detected pulse peak at the finger

Since pulsatile CSF flow was measured at the mid-C2 vertebral level, we assumed that measured pulsatile flow should sum to zero over the cardiac cycle due to the oscillatory nature of CSF flow dynamics with approximately zero net flow present in the spinal subarachnoid space over acute time scales [17, 18]. In practice, this was not observed, due to the presence of eddy currents and background phase offsets in the PCMR scan [19]. Therefore, we specified a subject-specific flow rate offset to force the net pulsatile CSF flow to be zero over each cardiac cycle as has been applied in previous studies [20–23]. The average required offset magnitude was 0.22 ± 0.34 mL/s. Similar to previous publications, we computed CSF flow volume as half of the integral of the absolute value of pulsatile CSF flow rate over the cardiac cycle. Numerical integration was performed using the trapezoid rule (Fig. 3) [24].

**Fig. 4:**
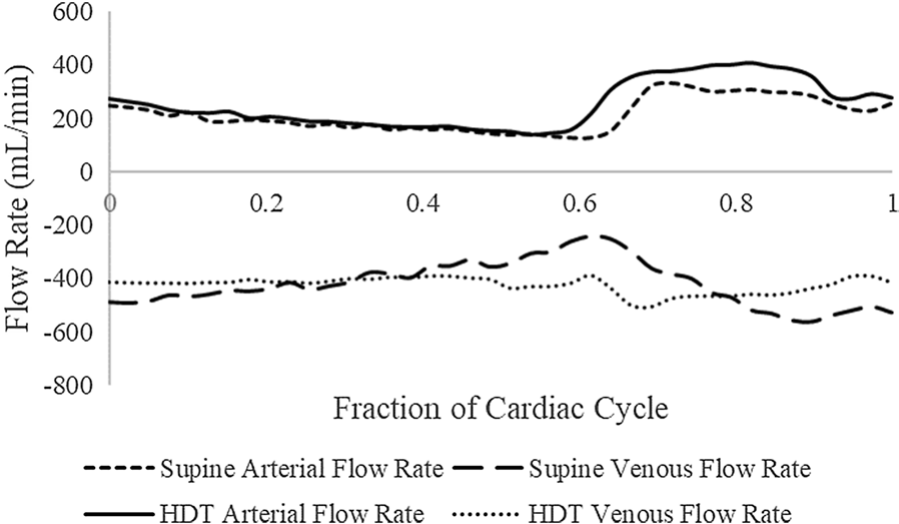
Representative arterial and venous flow rates vs. time over a cardiac cycle for a single subject for the right internal carotid artery and the right internal jugular vein. Negative values indicate flow towards the heart. The explanation for the late location of the systolic peak in this figure is the same as for Fig. 3

Instantaneous flow rates (mL/min) at 40 time points throughout the cardiac cycle were determined in the veins, arteries, and CSF space using the ROI area and corresponding average velocity measurements as determined by the FLOW program (Figs. 3 and 4).

Our primary outcome measures were: average flow rate over the cardiac cycle, systolic peak flow rate, peak-to-peak flow pulse amplitude (PtPPA), flow volume per cardiac cycle, and cross-sectional area (CSA) for arteries, veins, and the spinal subarachnoid space (Fig. 3). These specific variables were chosen to allow direct comparison with prior HDT studies (average systolic peak flow, flow volume/cycle, CSA) as well as due to their prior use in describing changes in intracranial CSF pulsations (PtPPA) [6, 12, 13, 25]. These outcome measures were determined from a combination of ROIs on the PCMR cine magnitude images (providing CSA) as well as measured velocity through that area.

### Statistical analysis

SAS version 9.4 and Microsoft Excel were used to conduct all data analyses. Normality for all study variables was assessed with a Shapiro-Wilk test. All outcome measures apart from venous cross-sectional area passed the Shapiro-Wilk test and were therefore analyzed through parametric methods, namely by paired two-tailed t-tests (supine vs. HDT). The comparisons in these statistical tests were conducted on each individual in a paired manner, comparing before and after HDT. The statistical significance of the difference in venous cross-sectional area due to HDT was accordingly analyzed through the non-parametric Wilcoxon signed-rank test. Differences were considered significant at p-value < 0.05.

## Results

Fifteen healthy subjects (7 males, 8 females; mean age ± SD: 29.5 ± 12.1 yrs.) participated in the study. Vital signs revealed no significant difference between heart rates or systolic and diastolic blood pressure before and after HDT (Table 1).

**Table 1:** Change in vital signs due to HDT (n = 15)

Vital Sign	Average Pre-HDT	Average Post-HDT	*p*-value
HR (bpm)	60 ± 9	59 ± 6	0.590
SBP (mmHg)	110 ± 14	116 ± 10	0.442
DBP (mmHg)	64 ± 12	71 ± 7	0.086

HDT, head-down tilt; HR, heart rate; SBP, systolic blood pressure; DBP, diastolic blood pressure

 Venous blood flow variables showed an increase in the internal jugular vein cross-sectional area from baseline to HDT (Table 2). In terms of arterial blood flow variables, changes in average blood flow rate, systolic peak flow rate, and PtPPA_art_ all reached statistical significance (Table 2). Finally, CSF flow variables showed a statistically significant decrease in systolic peak flow rate and PtPPA_csf_. (Table 2).

**Table 2:** Changes in cerebrovascular flow dynamics from baseline (supine position) to HDT (n = 15 subjects)

	Arterial blood flow					Venous blood flow				CSF flow			
	Baseline ± SD		HDT ± SD	%Δ	p-value	Baseline ± SD	HDT ± SD	%Δ	p -value	Baseline ± SD	HDT ± SD	%Δ	p-value
Average flow rate (mL/min)	867 ± 172		819 ± 177	−6 %	0.040*	−551 ± 214	−572 ± 157	+ 4 %	0.30	–	–	–	–
Systolic Peak flow rate (mL/min)	570 ± 108		522 ± 102	−8 %	0.040*	−474 ± 384	−576 ± 222	+ 22 %	0.24	−234 ± 84	−186 ± 66	−21 %	0.0088*
Diastolic Peak flow rate (mL/min)	444 ± 114		408 ± 90	−8 %	0.20	−498 ± 240	−492 ± 144	−1 %	0.93	138 ± 35	126 ± 52	−9 %	0.26
PtPPA (mL/min)	510 ± 126		450 ± 126	−12 %	0.020*	−456 ± 276	−486 ± 264	+ 7 %	0.24	372 ± 108	312 ± 102	−16 %	0.00098*
Flow volume/ cardiac cycle (mL/cardiac cycle)	15 ± 2.8		14 ± 3.4	−7 %	0.43	10 ± 4.6	10 ± 2.4	0 %	0.99	0.77 ± 0.20	0.68 ± 0.16	−12 %	0.10
CSA (mm^2^)	41 ± 7.6		43 ± 8.8	+ 5 %	0.32	82 ± 31	94 ± 40	+ 15 %	0.0050*	–	–	–	–

Asterisks indicate statistical significance

HDT, head-down tilt; CSF, cerebrospinal fluid; PtPPA, peak-to-peak pulse amplitude; CSA, cross-sectional area

## Discussion

Our hypothesis was that microgravity, as simulated through acute application of 15° HDT, would result in an increase in cardiac-related pulsatile CSF flow. Our results are inconsistent with this hypothesis, instead showing that HDT was significantly associated with a decrease in pulsatile CSF flow at the mid-C2 vertebral level as manifested by reduced systolic peak flow rate and PtPPA_csf_. In addition, HDT was significantly associated with decreased cerebral arterial average flow rate, systolic peak flow rate, and PtPPA_art_. Finally, a significant increase in jugular venous cross-sectional area was also observed.

Prior studies have examined cerebral flow dynamics under HDT. Marshall-Goebel et al., using 9 healthy male volunteers, found a decrease in both arterial and venous flow rate variables as well as an increase in venous cross-sectional area (CSA) from baseline to HDT (62 mm^2^ to 97 mm^2^), suggestive of increased venous pressure [13]. Ishida et al., using 15 healthy volunteers, found increases in venous CSA (36 mm^2^ to 54 mm^2^), decreases in arterial inflow, increases in venous outflow, and no significant changes in CSF flow volume/cycle or systolic velocity [12]. These observed changes in venous CSA under ground-based HDT are consistent with ultrasound studies performed during spaceflight that demonstrate comparable increases in venous CSA in subjects exposed to microgravity, which lends validity to the results of these HDT protocols [26, 27]. These prior studies collectively corroborate our findings of increased venous CSA and decreased arterial inflow. Finally, we observe a decrease in pulsatile CSF flow not examined in these prior HDT studies. The methods described in this work may be useful to understand CSF flow pulsatility increases that may be present after prolonged strict HDT.

Our findings of increased jugular venous cross-sectional area suggest increased cerebral venous pressure. Venous pressure has been shown by Holmlund et al. to be predictive of changes in ICP with increases in venous CSA correlating with increases in ICP [28]. These findings are, however, tempered by one study that showed that measured ICP and central venous pressure both decreased during acute (< 1 minute) episodes of zero G in parabolic flight, which differs from our findings of increased central venous pressure during HDT [29]. This difference may be due to the duration (< 1 min vs. longer duration HDT) or the mode (parabolic flight vs. HDT) of gravitational change.

To help interpret our CSF results, it is useful to recall several aspects of CSF physiology under normal gravity conditions, specifically the coupling of CSF and cerebral blood flow dynamics. CSF is produced in the choroid plexus of the brain’s ventricles and slowly circulates into the spinal and cranial subarachnoid spaces [30]. It is then absorbed into the venous system via arachnoid granulations, primarily near the dural venous sinus [30]. An increase in venous pressure can alter the pressure gradient across the arachnoid granulations, leading to reduced CSF absorption and an increase in total intracranial CSF volume [4]. However, a decrease in CSF absorption is not expected under acute HDT since any rise in venous pressure is likely counterbalanced by a similar rise in ICP. Our study did not directly measure change in intracranial CSF volume or compliance under acute HDT. Thus, we are not able to conclude how the observed decrease in CSF flow amplitude related to change, if any, in intracranial compliance.

As noted above, CSF flow from the brain has significant contribution from cardiac related pulsation, with cerebral arterial flow pulsations having a positive contribution to CSF flow pulsations [8]. SANS may involve perturbations to CSF flow associated with mild increases in ICP given the pathological findings of optic disc edema, globe flattening, and decreased visual acuity that are also present in Earth-bound conditions of CSF imbalance such as idiopathic intracranial hypertension [2, 31]. CSF dynamics can be altered by changes in production, flow, or reabsorption. However, the former and latter occur slowly, and thus are unlikely to be relevant in the setting of an acute HDT study.

Collectively, these results suggest that changes in cerebral arterial flow are a plausible cause for the observed decrease in CSF pulsatile flow. Because the elevated ICP expected in HDT would be expected to increase CSF flow pulsatility, we further suggest that the observed decrease in arterial pulsatility was a dominant factor leading to the findings. Since we did not measure intracranial compliance, it is not possible to determine the relative importance of intracranial compliance change with respect to CSF flow pulsatility under acute HDT .

Apart from the pathophysiological alterations suggested by the measurements in our study, these data are useful inputs for models seeking to simulate volume and pressure alterations in the head and eye in microgravitational settings. Prior studies have relied on blood and aqueous humor dynamics as inputs to models, and we anticipate that these results will be similarly useful for models seeking to replicate the pathophysiology of SANS [9].

The limitations of our study included the fact that our recorded venous outflow did not account for the entirety of arterial inflow, indicating missing venous collateral changes in flow. Jugular venous flow, as opposed to venous flow through other vessels such as the vertebral venous system, increases from an erect to supine position [32]. As a result, changes in body position from upright to supine to HDT could have effects on collateral venous flow that went unmeasured in our study. In addition, observed changes of flow in the jugular veins from supine to HDT in our study could plausibly be a result of the increased jugular recruitment seen in changes in body position from upright to supine [32]. We focused on the jugular veins in our study since they were the only veins that could be consistently identified in the images, but not being able to account for changes in collateral venous flow and their significance is a limitation.

A primary limitation of our study was the use of HDT as a proxy for microgravity. Although HDT has been utilized in many previous studies to simulate microgravity, it cannot reproduce other spaceflight-related nongravitational factors such as alterations to fluid and electrolyte balance, cardiovascular and pulmonary function, and metabolism, and therefore the generalizability of any findings to spaceflight has these inherent limitations [11, 33, 34]. An additional limitation includes the duration of the study. As previously mentioned, ocular symptoms in astronauts increase in prevalence with duration of spaceflight, and a 30-minute HDT analog captures only acute changes. Moreover, an ideal angle for HDT has not been established, with a range of angles from 6° to 15° being used. The 15° angle used in our study may have been too steep and we cannot ensure that our CSF findings would have been replicated with a lower degree of HDT. A study using a 15° HDT protocol to assess venous jugular blood flow before, during, and after spaceflight on International Space Station crew members demonstrated that venous CSA increased from sitting to supine to HDT with a similar magnitude of change between those positions preflight and postflight, although HDT in both of those settings overestimated increases in venous CSA found in-flight [35]. However, our venous and arterial changes from baseline to 15° HDT are corroborated by prior studies at these lower degrees of HDT, which lends support to the validity of our CSF findings at 15° [12, 13, 25].

Our scan protocol did not specify a defined stabilization period prior to the initial supine MRI scan. However, each volunteer was positioned in the supine position for at least 20 minutes before the scan used for evaluating flow. An additional limitation was that, due to time constraints, we did not repeat the scan protocol afterwards again in the supine position to assess return to baseline. We also were not able to monitor CO_2_ during our scan protocol and so cannot correlate our findings with any such changes in a patient’s breathing while undergoing these positional changes. Finally, the radiofrequency coil required that the head be at a 0° orientation above the neck. Excessive neck flexion has been associated with increased ICP and therefore could be a potential confounder of our findings [36].

In terms of limitations in statistical analysis, we chose not to apply a Bonferonni correction for multiple comparisons (due to the multiple variables in our study) so as to not make a type II error more likely. However, a lack of correction for multiple comparisons does increase the likelihood of a type I error. As a result, chance associations may exist, and our positive findings should be replicated. A final limitation includes the lack of well-defined minimal clinically important differences (MCIDs) in our flow variables with respect to SANS.

Future studies should compare arterial, venous, and CSF flow variables in astronauts pre- and post-flight, particularly in those experiencing SANS symptoms in order to generate MCIDs. Non-invasive measurements of these flow variables in astronauts during spaceflight would also be ideal if permitted by technology. In addition, comparisons of HDT variables with imaging from astronauts would help validate HDT as a tool for further investigation of physiological changes to the head in microgravity, which would be critical to justify future studies examining specific populations of interest such as women, minorities, various age groups, or even children to create more individualized risk profiles and predictive models.

In conclusion, we have demonstrated that acute application of 15° HDT to simulate microgravity was associated with alterations in intracranial blood flow and spinal CSF flow dynamics, specifically a reduction in CSF pulsatile flow variables. When combined with our observed decreases in cerebral arterial flow variables as well as a significant increase in jugular venous cross-sectional area, these findings collectively suggest that decreases in cerebral arterial flow were the principal drivers of our observed decreases in CSF pulsatile flow.

## Data Availability

The datasets used and analyzed during this study are available from the corresponding author on reasonable request.
